# Substitution rate and natural selection in parvovirus B19

**DOI:** 10.1038/srep35759

**Published:** 2016-10-24

**Authors:** Gorana G. Stamenković, Valentina S. Ćirković, Marina M. Šiljić, Jelena V. Blagojević, Aleksandra M. Knežević, Ivana D. Joksić, Maja P. Stanojević

**Affiliations:** 1 Department of Genetic Research, Institute for biological research “Siniša Stanković”, University of Belgrade, 142 Despot Stephan Blvd, 11060 Belgrade, R Serbia; 2 Institute for Microbiology and Immunology, School of Medicine, University of Belgrade, 1/1 Dr Subotića St, 11000 Belgrade, R Serbia; 3 Clinic of Obstetrics and Gynecology “Narodni front”, 62 Kraljice Natalije St, 11000 Belgrade, R Serbia

## Abstract

The aim of this study was to estimate substitution rate and imprints of natural selection on parvovirus B19 genotype 1. Studied datasets included 137 near complete coding B19 genomes (positions 665 to 4851) for phylogenetic and substitution rate analysis and 146 and 214 partial genomes for selection analyses in open reading frames ORF1 and ORF2, respectively, collected 1973–2012 and including 9 newly sequenced isolates from Serbia. Phylogenetic clustering assigned majority of studied isolates to G1A. Nucleotide substitution rate for total coding DNA was 1.03 (0.6–1.27) x 10^−4^ substitutions/site/year, with higher values for analyzed genome partitions. In spite of the highest evolutionary rate, VP2 codons were found to be under purifying selection with rare episodic positive selection, whereas codons under diversifying selection were found in the unique part of VP1, known to contain B19 immune epitopes important in persistent infection. Analyses of overlapping gene regions identified nucleotide positions under opposite selective pressure in different ORFs, suggesting complex evolutionary mechanisms of nucleotide changes in B19 viral genomes.

Human parvovirus B19 (B19) is a widespread member of the family Parvoviridae that causes a variety of clinical manifestations, from asymptomatic to persistent infection associated with different autoimmune diseases^1^,^2^. As all parvoviruses, B19 depends on the S phase of the host cell for replication, resulting in its wider tropism for fetal tissues and much narrower tropism range for adult cells^2^.

B19 virions are nonenveloped icosahedral particles with a linear single-stranded DNA genome of approximately 5600 bp. At both ends of the B19 genome, there are identical inverted terminal repeats of 383 nt in length. Coding sequence of the B19 genome (≈4.8 kb) is divided in two main open reading frames (ORFs), one encoding the nonstructural protein (NS1) and the other encoding both major VP2 and minor VP1 structural proteins^1^,^3^. The only difference between VP1 and VP2 is in the N terminal “unique region” (uVP1) composed of 227 amino acids. VP2 builds 95% of the capsid containing self-assembly domains that lead to formation of highly stable particles. The role of VP1 is not essential for capsid formation, but its uVP1 region is critical for virus entry via phospholipase A2 (vPLA2) domain^4^. NS1 is the main non-structural multi-functional protein, with the central role in controlling viral DNA replication and transcription^3^,^5^,^6^. In addition, NS1 induces cell cycle arrest, apoptosis and modulation of host innate immunity^7^,^8^,^9^.

B19 infection induces long-lasting antibody and cellular responses^10^. Viremic phase onsets in the first week of infection and reaches extremely high viral concentrations of 10^10^ to 10^13^per mL of plasma/serum^3^,^11^. Viremia declines with appearance of IgM antibodies against linear and conformational epitopes of viral capsid proteins VP1 and VP2, with the peak levels during the third weeks after infection. Majority of studies found that, irrespective of the underlying disease, NS1-specific IgG antibodies appear late in infection, principally in patients who develop persisting viremia^10^,^12^.

B19 sequences cluster into three genotypes, further divided to subtypes. Currently, in addition to the worldwide predominant genotype 1, with subgenotypes 1A and 1B, genotypes 2 and 3 with two subtypes 3a and 3b are identified^13^,^14^. All genotypes have similar functional, structural and immunological characteristics and comprise the same serotype^15^.

Members of the family Parvoviridae are characterized by high genetic diversity with substitution rates in the range of 1–2 × 10^−4^ per site per year, similar to those of ssRNA viruses^16^. So far, B19 substitution rate has been estimated on partial NS1 and VP1 gene sequences for genotypes 1 and 3, with two studies investigating near full-length B19 genome, albeit including limited number of sequences^14^,^17^,^18^,^19^. Lately, the number of B19 genome sequences deposited in DNA sequence databases has largely increased. We aimed to reevaluate B19 genome variability data and phylogenetic relations in the most prevalent B19 genotype 1, using near complete coding DNA (cDNA) sequences currently present in the GenBank database, together with newly acquired B19 sequences from Serbia, generated for this study. Further, with different codon-based maximum likelihood methods we analyzed the extent of selection pressure on particular genes or codons, aiming to investigate the impact of natural selection to high B19 substitution rate.

## Results

### Phylogenetic analysis

**Figure 1:**
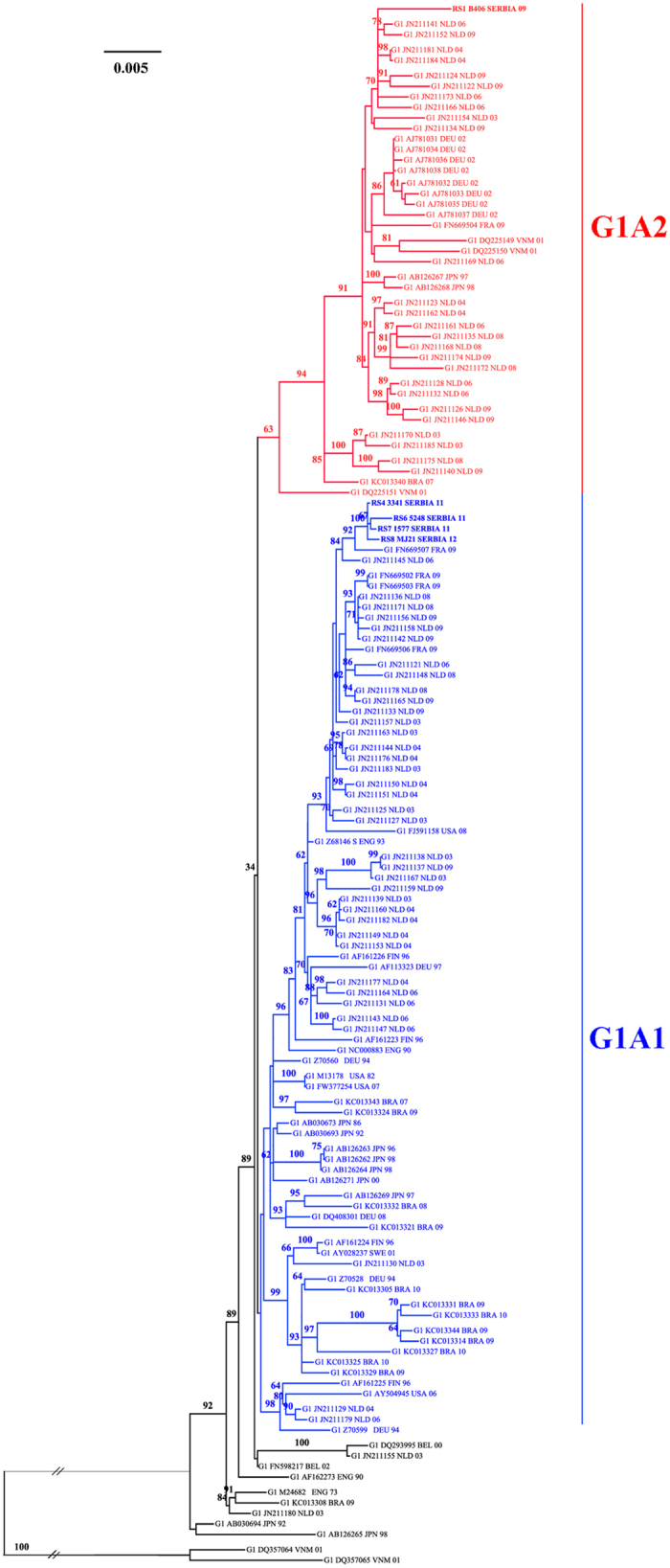
Phylogeny of B19 genotype 1 complete coding region. Legend for Fig. 1: 133 studied isolates are presented with GenBank accession number, three letters ISO country code and year of isolation. The ML tree was constructed using PAUP, under the best-fit substitution model as determined by jModeltest, TIM3 G + I (CI 95%). Bootstrap values with 1000 replicates were obtained using IQTREE online software.

The results of phylogenetic analysis were consistent, by all the applied methods. Reconstructed phylogenetic tree revealed clustering of genotype 1A isolates into two large lineages, containing 122/133 (93.13%) of all analyzed isolates (Fig. 1), one consisted of 80/122 and another one of 42/122 isolates, corresponding to clusters 1A1 and 1A2, respectively. Remaining 9/133 isolates, sampled in a large time span from 1973 to 2003, formed 4 additional distinct small clusters. Local, Serbian isolates RS2 to RS5 formed a sub-clade in the major cluster of subtype 1A1, whereas isolate RS1 was found separated in second major cluster 1A2.

Average nucleotide distance in the whole analyzed near complete cDNA dataset of 133 B19 genotype 1 isolates was 0.014, s.d. = 0.009. Nucleotide distance between subgenotypes 1A and 1B was 0.055, s.d. = 0.003. Intragroup nucleotide distance for 1A subgenotype was 0.013, s.d. = 0.005.

### Substitution rates

**Figure 2:**
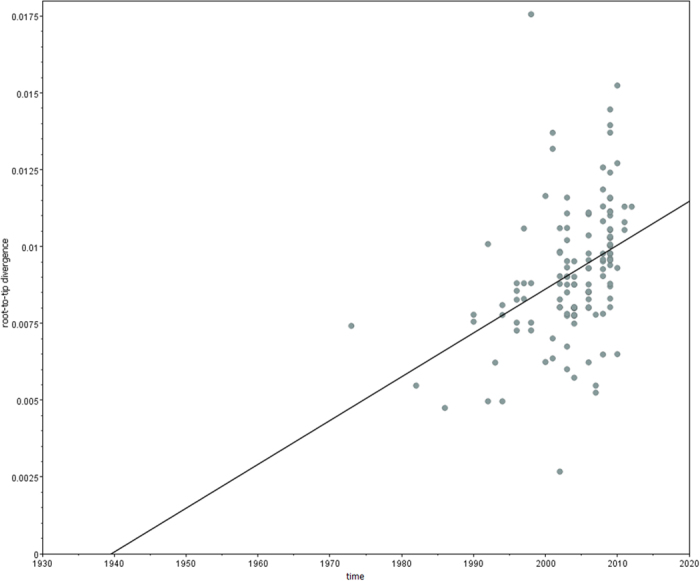
Root-to-tip regression analyses of B19 cDNA sequences used for substitution rate callculation. Legend for Fig. 2 Linear regression plots of the root-to-tip divergence (nucleotide substitution/site) against sampling year; ML phylogenetic tree constructed in PhyMl v.3.0 was imported for analyses.

Root to tip linear regression analysis revealed sufficient temporal structure of the collected dataset (R2 = 0.15, Correlation coefficient = 0.39 (Fig. 2). Evolutionary rate was calculated on near complete cDNA dataset and on the same dataset partitioned based on open reading frames (ORF1 and ORF2). Results are shown in Table 1.

**Figure 3:**
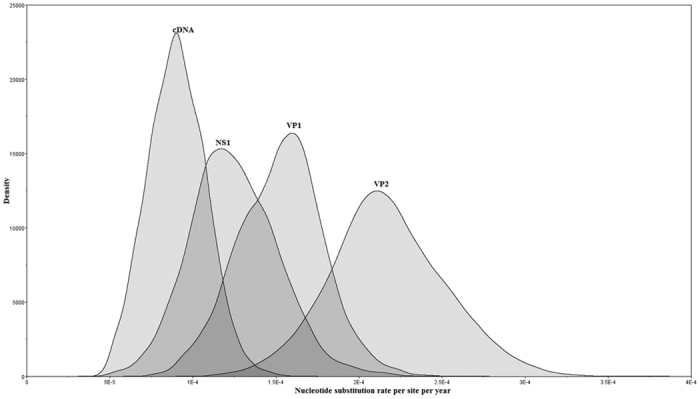
Evolutionary rates for B19 cDNA and genome partitions. Legend for Fig. 3: Marginal distributions of the rates from the different genome partitions; analysis was performed using BEAST under strict clock model.

The highest substitution rate was observed for VP2, the functional part of VP1, 2.32 × 10^−4^ substitutions/site/year, respectively. The values of substitution rates for NS1 and VP1 were very close and significantly lower than VP2, yet higher than the rate of cDNA (Table 1). Marginal distributions of the rates from the different genome partitions for the strict clock data are presented in Fig. 3, showing that indeed they overlap for the regions NS1 and VP1 mutually and with both cDNA and VP2 on either side of the graph. However, this is not the case with marginal distributions for cDNA and VP2. Hence, we conclude that the rate for VP2 is indeed significantly different compared to cDNA. Since the 95% HPD interval for nucleotide substitution rate in uVP1 was found to be rather large, encompassing the values for other partitions, highlighting the uncertainty in the rate estimate in this region, we excluded this region from the comparisons.

### Natural selection

Regarding two main ORFs of B19 genome, overall selection pressure, measured as the mean ratio of nonsynonymous (dN) to synonymous substitutions (dS) per site (dN/dS) was 0.150 and 0.087 for NS1 and VP1, respectively.

**Figure 4:**
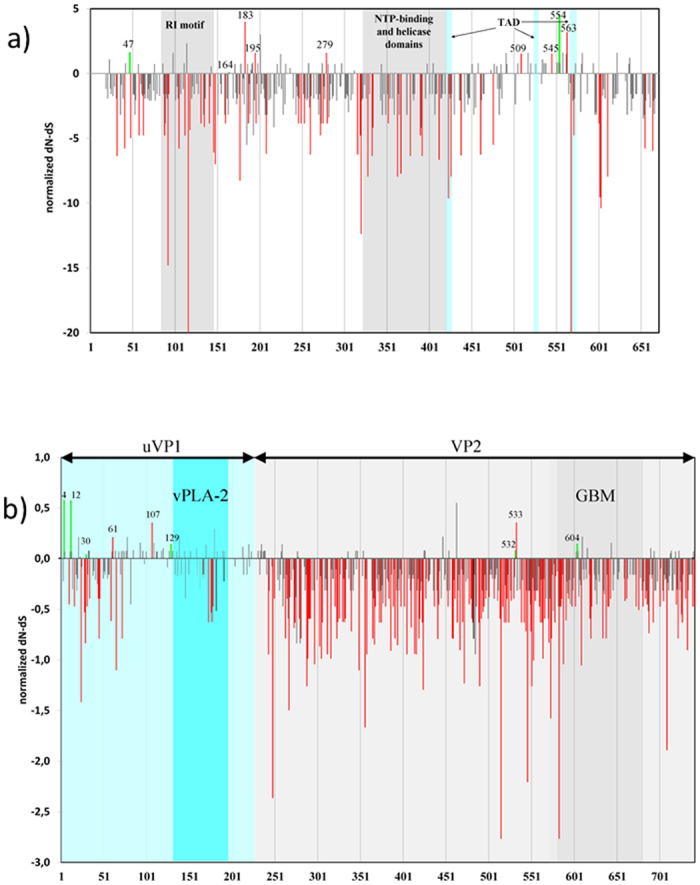
Selection pressure on two major B19 genes encoding NS1 and VP1 proteins. Legend for Fig. 4: Red bars - non-neutral selection identified as pervasive in any of used statistical approach with P < 0.1; green bars - non-neutral selection identified as episodic by MEME with P < 0.1; X-axis represents codon positions; normalized dN-dS value obtained by SLAC method presented on Y-axis. Fig. 4a) NS1 gene (codons 18–671), particular NS1 protein domains highlighted: RI motif - replication-initiator motif: codons 79–147; NTP-binding and helicase domains: codons 320–416; putative transactivation domains: TAD1: codons 416–424; TAD2: codons 523–531; TAD3: codons 566–576; Fig. 4b) VP1 gene, including distinct protein parts uVP1 (codons 1–227) and VP2 (codons 227–742) and domains: vPLA2 (codons 130 to 195); GBM - globoside-binding motif (codons 577–677).

Majority of variable codons in both NS1 and VP1 genes were found to be under strong negative selective pressure or neutrally evolving (P < 0.1) (Fig. 4). Detection of positively selected sites by different analytical algorithms applied is presented in the Supplementary Table S2. In short, 10 codons in VP1 and 9 codons in NS1 were identified under diversifying selection (Supplementary Table S2, Fig. 4). Of those, 3 sites in VP1 and 1 position in NS1 were identified as positively selected by two or more analysis methods.

Selection pressure analysis on B19 genes coding for small protein products of 7.5 kDa, 9 kDa and 11 kDa estimated mean dN/dS of 0.297, 0.760 and 0.463, respectively, which is substantially higher compared to large proteins (Supplementary Table S2).

In particular, we compared traces of natural selection in overlapping B19 genes that are expressed in different reading frames. Substitution T2276C is non-synonymous in the NS1 gene (F554 → L) with indication of positive selection (FEL and IFEL, P < 0.05), whereas the same substitution is synonymous when expressed as the first nucleotide of codon 65 (TTG → CTG) in the 7.5 kDa gene, where it is found to be subjected to purifying selection force (P < 0.05, Supplementary Table S2). Transition T3061G was positively selected in codon 63 of 9 kDa (TTG → TGG, L63W, FEL P < 0.05), whereas in uVP1 it induces synonymous substitution with negative selection force on codon 146 (GTT → GTG, P < 0.1 in FEL and IFEL). Similarly, non-synonymous substitution G2916A, in the 9 kDa gene (GCA → ACA, A15T) is found under positive selection (FEL P < 0.05), while in the same time, it is found under neutral selective force in the uVP1 gene (AGC → AAC, S98N, Supplementary Table S2).

To elucidate a non-random usage of synonymous codons for specific amino acids we calculated Relative Synonymous Codon Usage (RSCU) values, which are presented as the observed frequency of a codon, divided by its expected frequency under the assumption of equal codon usage^20^. RSCU values, calculated for two ORFs of B19 genome, showed that some codons were favored in both major genes (Supplementary Table S3). For NS1, this is the case for synonymous substitutions in codons with the highest value of negative selection (normalized dN-dS < -10), such as 116 (GTG → GTA, RSCU are 1.06:1.37), 320 (AAG → AAA, RSCU are 0.51:1.49) and 586 (CAG → CAA, RSCU are 0.86:1.14). In VP1, the highest value of negative selection (normalized dN-dS < -2) for synonymous substitutions was found for codons: 515 (TTC → TTT, RSCU are: 0.05:1.95), 546 (GGT → GGA, RSCU are 0.95:1.81) and 583 (CAG → CAA, RSCU are 0.86:1.14).

Natural selection and substitution rate analyses of B19 genome, as evolutionary parameters, showed that substitution rate of the ORF2 region, coding for VP1 and VP2 structural proteins was higher compared to ORF1, albeit with stronger purifying selection (VP1 = 1.64 vs. NS1 = 1.36 substitutions/site/year x 10–4; mean dN/dS for VP1 = 0.087 vs. NS1 = 0.150). In the partitioned analyses of VP1, uVP1 region was characterized by the highest dN/dS ratio, albeit with the lowest substitution rate, compared to genes coding for large B19 proteins (Table 1, Fig. 4).

## Discussion

We studied substitution rate and natural selection in parvovirus B19 genotype 1.

Parvovirus B19 genotype 1, the most frequent worldwide, is known to be divided into subgenotypes: the predominant 1A and rarely found 1B^1^,^2^. So far, only two complete cDNA subgenotype 1B sequences originating from Vietnam are present in the GenBank (DQ357064 and DQ357065). Besides, Barros de Freitas *et al*. detected several subgenotype 1B isolates in patients with hematological disorders from Brazilian Amazon region, based on 476 nt in ORF2 genome region^21^. The same phylogenetic study described for the first time two clear clades in subgenotype 1A, designated as 1A1 and 1A2. This clustering within subgenotype 1A was confirmed by phylogenetic analysis based on 446 nt in NS1 gene region, also in Brazilian isolates^19^, and in isolates collected in the Netherlands based on almost complete cDNA genome sequences^22^.

Our phylogenetic analysis of B19 genotype 1 is in line with previous findings of two main clades within subgenotype 1A, subtypes 1A1 and 1A2. However, the bootstrap support for the key nodes is less than 70%, indicating insufficient phylogenetic resolution to firmly support the existence separate subtypes. Of note, we found 9 isolates clustering within the 1A genotype, yet outside the two presumed 1A subtypes (Fig. 1). Gathering of additional sequence data would help to elucidate the true topology of B19 genotype 1 phylogeny and the distinct subtypes and clades, as proposed by some investigators^19^,^22^.

Generally, phylogenetic clustering in our analysis did not tend to correlate to either collection time or geographical location of sequence origin. However, some isolates from the same country and close collection date, did cluster together, e.g. isolates from France (FN669503, FN669504, FN669506, 2009), Germany (AJ781031 to AJ781038, 2002), Brazil (KC13327, KC13344, KC13331, KC13333-2009 to 2010) and Japan (AB126262 to AB126264, 1996 to 1998, AB126271-2000, AB030673-1986, AB030693-1992). On the other hand, isolates from the Netherlands, the largest collection of 62 almost complete B19 sequences from the same country, collected in the period 2003 to 2009, were found dispersed along both clades 1A1 and 1A2 (Fig. 1). Similarly, newly obtained isolates in our study were found scattered within the subtype 1A1 (RS4 and RS6 to RS8, collected in 2011 to 2012) and the isolate RS1, collected in 2009, was the only one that clustered in the subtype 1A2. Generally, existing B19 molecular epidemiology studies found subtype 1A1 to be more prevalent than 1A2, although these two subtypes co-exist around the world. However, clear functional (biological) difference between the subtypes in ability to cause different symptoms has not been found^19^,^22^,^23^.

Our estimation based on near complete cDNA of genotype 1A sequences, isolated in different parts of the world over time span of 40 years revealed the rate of accumulation of nucleotide changes in the B19 cDNA of 1.03 ± 0.1 × 10^−4^ substitutions/site/year. Previous studies, based on the alignment of a similar length (4216 nt, positions 654 to 4869 nt) but much smaller in size, estimated B19 evolutionary rate at 1.83 × 10^−4^ substitution/site/year for genotype 1, and 2.0 × 10^−4^ substitution/site/year for genotype 3^14^,^17^.

Recent studies of viral evolutionary dynamics have shown that evolutionary rate of ssDNA viruses is similar to the one of ssRNA viruses. Besides human parvovirus B19, substitution rate in the order of magnitude of 10^−4^ was shown for other ssDNA viruses such as the circovirus SEN-V and plant geminivirus Tomato yellow leaf curl virus^16^,^24^. Substitution rate depends on many factors concerning viral life cycle, such as mutation rate, generation time, transmission and natural selection^16^. Mutation rate of B19 virus, in the sense of real time measurement of nucleotide changes in biological systems has not been measured so far. It could be expected to be high, in view of single stranded architecture of B19 genome, of small size and high replication turnover in the acute faze of infection, when viremia frequently reaches 10^10^–10^13^ genome equivalents/mL^11^. Since fixation of mutation is directly influenced by forces of natural selection, we compared substitution rate and selection pressure on discrete gene regions. Our findings (Table 1) are in line with previous reports by Shackelton and Holmes that estimated substitution rates for both ORF1 and ORF2 to be in the same order of magnitude as complete coding DNA, however, substitution rate of ORF2 was higher compared to ORF1^17^.

In our study selection analysis revealed negative selection to be in action on the most of the B19 genome. Functional aspects of viral proteins may significantly effect on selection pressure, limiting fixation of new mutations. As a major structural protein accounting for about 95% of the capsid, VP2 also contains the receptor-binding site i.e. globoside-binding motif from 577aa to 677aa^2^,^3^. Involvement of VP2 in capsid composition and cell entry determines numerous strongly negatively selected codons and weak positive selection. We identified codons under strong negative selection in other VP2 positions with known or presumed functional roles: non-synonymous substitution V21A close to the VP2 N-terminal end could be important in protein folding and capsid formation, whereas the basic motif (493KLGPRKATGRW503) found in the VP2 C-terminal region is known to be necessary for nuclear localization of viral particle^25^,^26^,^27^. Other codons (515, 546, 583 and 709) under highest negative selection pressure in VP2 contain synonymous substitutions, reflecting codon usage pattern as depicted by us or composition and gDNA packaging into the virion, as also speculated by Shackelton *et al*.^20^

Complete loss of virus infectivity in uVP1 null mutants exemplifies the crucial role of uVP1 protein in viral life cycle, in particular, enzymatic role of B19 phospholipase A2-like (vPLA2), encoded by uVP1 nt 3011–3208 (codons 130–195). This is reflected by full conservation of uVP1 motifs 130YXGXG134 and 153HDXXY157, found in our analysis^4^,^28^.

NS1 has a conserved architecture consisting of: N-terminal DNA-binding/nickase and endonuclease domain^5^; central region with conserved NTP-binding and helicase/ATPase motifs^29^; and the unique C-terminal region suggested to interact with different proteins^18^,^30^. We found substantial portion of the NS1 protein to be under purifying selection, in particular regions implicated in its enzymatic functions. C-terminal end of NS1 has been previously shown to be highly polymorphic and involved in host-protein interactions and promoter trans-activation^18^,^30^. In our analysis, positive selection was absent from previously defined transactivation domains (TAD1 to TAD3)^31^, participating in disruption of cell cycle at the S phase.

In our analysis ten codons in VP1 and 9 codons in NS1 were identified under diversifying selection with any of the methods used, whereas only 3 sites in VP1 and 1 position in NS1 were identified as positively selected by two or more analysis methods, reflecting inherent limitations of the methods used to estimate evidence of selective pressure at the codon level. In their analysis of a smaller B19 dataset оf 38 sequences Shackelton and Holmes did not reveal any codons with positive selection^17^. However, it was shown that the ability of the employed approaches to identify codons under positive selection pressure greatly depends on the number of sequences used in analyses^32^.

Positions under positive selection in different parts of the ORF2 could reflect immune response as the main driver of natural selection, since the 3 identified positively selected VP1 codons (4, 12, 107) fall within known VP1 immune dominant epitopes (VP1-F1 (aa 2–100), VP1-F2 (aa 99–227))^33^,^34^,^35^.

A number of studies dating from the nineties have identified several B cell epitopes in VP2, conformational and linear, implicated mostly in the acute phase immune response^12^,^35^. Many amino acid substitutions in conformational epitopes do not affect antibody binding^33^, leading to the increased limit of viability (the so-called error threshold), resulting in selective forces being neutral^16^. Consequently, in spite of higher substitution rate in VP2 (uVP1 = 1.11 vs. VP2 = 2.32 substitutions/site/year x 10^−4^), stabilizing selection is more expressed in this region (mean dN/dS: uVP1 = 0.325, VP2 = 0.055). Notably, in our analyses, positively selected codons appeared rarely in the complete VP1 and VP2, characterized as episodic selection, contrary to uVP1 with diversifying substitutions, pivotal for long persistent infection. Signals of diversifying pervasive selection identified at NS1 positions 195 and 279 coincide with antigenic determinants known to be implicated in persistent infection^10^,^12^,^24^.

Genes coding for B19 small proteins (7.5 kDa and 9 kDa) are known to be the least variable in B19 genome^17^,^18^, as also shown in our analysis. So far, only few sequences of the 11 kDa have been deposited to public sequence databases. In this study, we contributed with 6 full sequences of 11 kDa sequences of B19 genotype 1, in addition to 21 previously exiting in the GenBank. Based on very limited dataset, this region is highly conserved with rare and mostly neutral substitutions.

Of note, we identified opposite action of natural selection on the same nucleotide positions in the overlapping genes, expressed in different reading frames. Sustainability of these polymorphisms in overlapping genes could depend on the influence of the nucleotide substitution on adaptive evolution of both proteins and their overall impact on viral fitness.

## Conclusion

Here, we present phylogenetic analysis of the largest dataset of 133 near complete coding B19 genotype 1 sequences analyzed so far. Substitution rate analysis confirmed high substitution rate of B19 DNA genome, comparable to RNA viruses, in the range of 10^−4^ substitution/site/year. Generally, negative selection was found in action on the most of the B19 genome, with diversifying selection operating at certain codon positions, located mainly in antigenic domains and consequently driven by immune response pressure. Complex mechanism of maintenance of genome variability is demonstrated by codon selection analyses in overlapping gene regions with selection in opposite direction in the same nucleotide positions.

Gathering of additional sequence data would help to elucidate parvo B19 genotype 1 evolution.

## Materials and Methods

### Patient samples

We collected 10 blood samples (with EDTA) from patients seropositive for B19 IgM and/or IgG, or having symptoms indicative of B19 infection. Four out of ten were serial samples from same individuals. All samples were collected during 2009–2012, after obtaining informed consent from the patients. Plasma was separated immediately (except for RS-1 that was frozen as whole blood), and stored frozen (−20 °C) prior to testing. The study was approved by the institutional ethical committee of the Clinical Center of Serbia. All experimental methods involving human participants were carried out in accordance with the relevant guidelines and regulations.

### B19 genome detection and sequencing

Viral DNA was extracted from 200 μL of plasma or blood using QIAamp MinElute Virus Spin Kit or QIAamp DNA Blood Virus Spin kit, respectively (QIAGEN GmbH, Germany), according to manufacturer’s instruction, and eluted with 60 μL of elution buffer. Complete cDNA was PCR amplified with primers listed in Supplementary Table S4. Amplicons were sequenced in both directions by BigDye Terminator v3.1 Cycle Sequencing Kit (PE Applied Biosystems, Foster City, CA) and sequences were basecalled and assembled by ABI softwares: Sequencing Analysis 5.1 and SeqScape software, v 2.5.

Nucleotide sequences were successfully retrieved from 8/10 analyzed patient samples: 5 near complete and 4 partial B19 genome sequences were obtained, designated RS1-10. Obtained B19 sequences were deposited in the GenBank under accession numbers KR005636- KR005644 (Supplementary Table S1). No unusual stop codons, frame-shifts, insertions or deletions were found in the obtained sequences, except for RS1 isolate, lacking the start codon for 11 kDa protein. Two sequence pairs, successively sampled from the same individuals, were recovered: isolates RS1 and RS2, retrieved during an exchange transfusion for fetal hydrops (RS1) and then from the mother after one year (RS2); isolates RS8 and RS9/RS10 collected within an interval of three weeks, from adult patient with migratory arthritis and pericarditis. Notably, there was no nucleotide divergence between isolates of the latter pair, whereas distance between the former pair was 2.6% (s.d. = 0.02), among the highest ones in collected genotype 1A isolates.

### Sequence datasets

B19 genotype 1 sequences present in the GenBank database covering ≈95% of genomic cDNA (from nt position 665 to 4851, numbering according to reference B19 isolate NC_000883.2) were collected, resulting in the total of 137 sequences. Only sequences with available collection time/place containing no deletions and insertions were included in the study (Supplementary Table S1A). For the analyses of selection pressure we analyzed codon-based alignments: for ORF1 and ORF2, with the total of 146 and 214 sequences, respectively, and additional 27 sequences for 11 kDa small protein within ORF2, deposited in the GenBank database (Supplementary Table S1B).

### Phylogenetic analysis

Multiple nucleotide sequence alignments were created of almost complete cDNA sequences and separately for two reading frames (ORF1 and ORF2), using CLUSTAL W, as implemented in MEGA 6 software^36^. The best-fit nucleotide substitution model for aligned sequences was determined by jModeltest 2.1.4 software^37^ using all 88 proposed models. Bayesian information criterion (BIC) was used to determine the model of nucleotide substitution that best fits the data for each of the subsets analyzed. Alignments were screened for recombination using RDP, GENECONV, Bootscan, SiScan recombination detection approaches as implemented in the program RDP4 v.4.36^38^. Recombination screening of the analyzed dataset of 137 B19 genotype 1 cDNA sequences (Supplementary Table S1A) detected 4 putative recombinants, that were excluded from further phylogenetic analyses (DQ225148, AB126266, KC013312 and AB126270).

Further phylogenetic analyses, construction of trees and nucleotide distance calculation were performed by both neighbor joining and maximum-likelihood approach implemented in Phylogenetic Analysis Using Parsimony (PAUP) version 4.0b10 software package^39^. Bootstrap support for the tree nodes of the reconstructed phylogenetic trees was calculated with 1000 replicates by IQTREE v. 1.1.0 software^40^. Bootstrap values exceeding 70% were considered significant.

To explore temporal structure of the sequences included in the analysis an exploratory root-to-tip linear regression was performed with TempEst v. 1.5, by importing ML phylogenetic tree constructed in PhyMl v.3.0^41^,^42^. This method performs a linear regression between the time of sampling of each tip and the genetic distance from the root.

### Substitution rates

The nucleotide substitution rates were estimated using Bayesian Markov Chain Monte Carlo (MCMC) approach implemented in BEAST v.1.8.3^43^. In order estimate the best fit evolutionary model, the analyses were initially performed under both strict and relaxed (uncorrelated exponential and uncorrelated lognormal) molecular clocks, with Bayesian skygrid as coalescent tree priors. The MCMC chain was run for 30,000,000 steps with parameter values sampled at every 3000 steps. Log marginal likelihoods were determined by generalized stepping stone sampling. The best fit model was chosen according to Bayes factor. The analysis under the chosen model was performed in two additional runs in 50,000,000 steps each, with sampling at every 1000 steps and the results were combined using LogCombiner 1.8.3 (implemented in BEAST) with 10% burn-ins removed from each run. The resulting log files were further explored in Tracer 1.6 to ascertain convergence of the chain and ESS values >200 for all parameters. The uncertainty in the parameter estimates were assessed by 95% HPD interval (Table 1). Marginal probability distribution test integrated in Tracer v.1.6 was applied for comparison data of mean nucleotide substitution rate for all analyzed genome regions.

### Estimation of evolutionary pressure

Selection pressure was analyzed in two B19 major proteins (NS1 and VP1) and three small proteins (7.5 kD, 9 kDa and 11 kDa protein), based on the alignments described above and in Supplementary Table S1B.

Evolutionary pressure was assessed using HyPhy software package implemented by the Datamonkey web-based facility (http://www.datamonkey.org)^44^. Overall selection pressure, measured as the mean ratio of nonsynonymous (dN) to synonymous substitutions (dS) per site (dN/dS), was estimated using four different likelihood approaches for analyzed datasets: the Single Likelihood Ancestor Counting (SLAC), Fixed-Effects Likelihood (FEL) internal branch Fixed-Effects Likelihood (IFEL) and Random-Effects Likelihood (REL) methods^44^. In addition, we used mixed effects model of evolution (MEME) that is capable of identifying instances of both episodic and pervasive positive selection at the level of an individual site^45^. For all the methods, Tamura-Nei model (TrN) or Hasegawa-Kishino-Yano (HKY85) were used as nucleotide substitution model. Separate phylogenetic tree for each analyzed partition was inferred by the neighbor-joining method (NJ) implemented in the HyPhy package available on the Datamonkey webserver. The diversity scores were considered to be significant at a confidence interval of p ≤ 0.1. Relative Synonymous Codon Usage (RSCU) values were determined by MEGA 6 software^36^,^46^,^47^.

## Additional Information

**How to cite this article**: Stamenković, G. G. *et al*. Substitution rate and natural selection in parvovirus B19. *Sci. Rep.* **6**, 35759; doi: 10.1038/srep35759 (2016).

## Supplementary Material

Supplementary Information

## Figures and Tables

**Table 1:** B19 genotype 1 nucleotide substitution rate.

Data set^a^	analyzed partitions (nt)^b^	Clock model^c^	Log Marginal Likelihood^d^	Nucleotide substitution rate (10^−4^ substitutions/site/year)	
				Mean ± S.E.	HPD
cDNA	665–4851	Relaxed exponential	−17913		
substitution model	TIM3 + I + G	Relaxed lognormal	−18250		
		Strict	−16535	**1.03** ± **0.01**	0.56–1.27
NS1	667–2631	Relaxed exponential	−8935		
substitution model	TIM3 + I + G	Relaxed lognormal	−8925		
		Strict	−7965	**1.36** ± **0.01**	0.73–1.75
VP1	2624–4851	Relaxed exponential	−10604		
substitution model	TIM3 + I + G	Relaxed lognormal	−10606		
		Strict	−9658	**1.64** ± **0.01**	1.00–2.00
uVP1	2624–3305	Relaxed exponential	−3836		
substitution model	TPM3 + I + G	Relaxed lognormal	−3837		
		Strict	−2847	**1.11** ± **0.03**	0.04–3.10
VP2	3305–4851	Relaxed exponential	−8448		
substitution model	TrN + I + G	Relaxed lognormal	−8476		
		Strict	−7486	**2.32** ± **0.01**	1.53–2.86

^a^131 isolates used in analyses listed in Table S1a.

^b^numbered according to the reference sequence NC_000883.2.

^c^coalescent tree prior for all analyses was Bayesian Skygrid.

^d^Log Marginal Likelihood obtained using Stepping Stone Sampling; Abbreviations: HPD - Highest Posterior Density interval contains 95% of posterior probability distribution of nucleotide substitution rate, S.E. – standard error of mean.
