# Supplementary material for: Substitution rate and natural selection in parvovirus B19
Source: Sci Rep. 2016 Oct 24;6:35759. doi: 10.1038/srep35759 (PMC5075947; doi:10.1038/srep35759)
Supplement: Supplementary Information [file srep35759-s1.doc]

**Supporting information:**

**Substitution rate and natural selection in parvovirus B19**

Gorana G. Stamenković, Valentina S. Ćirković, Marina M. Šiljic, Jelena V. Blagojević, Aleksandra M. Knežević, Ivana D. Joksić, Maja P. Stanojević

**Supplementary Table S1 B19 genotype 1 genome sequences used for evolutionary analyses**

Legend for Supplementary Table S1: S1A. B19 sequences used for phylogenetic analyses and substitution rate calculation; S1B. B19 sequences used for codon selection analyses.

1A. Isolates used for phylogenetic analyses and substitution rate calculation

| Location, collection year and GenBank accession number (isolation name) |
| --- |
| **Japan** *(n=13)* |
| 1986: AB030673 (N8) |
| 1992: AB030693 (Mi) |
| 1992: AB030694 (Rm) |
| 1996: AB126263 (AN28)1997: AB126266 (AN40), AB126267 (AN41) |
| 1998: AB126262 (AN23), AB126264 (AN30), AB126265(AN34), AB126268 (AN56), AB126269 (AN66), AB126270 (AN85) |
| 2000: AB126271 (B18-AN87) |
| **Finland** *(n=4)* |
| 1996: AF161223-6 (Kati1-4) |
| **Germany** *(n=13)* |
| 1994: Z70560 (1/I), Z70528 (2/II), Z70599 (Sp2) |
| 1997: AF113323 (SLE) |
| 2002: AJ781031-8 |
| 2008: DQ408301 (BN30.1) |
| **Sweden** *(n=1)* |
| 2001: AY028237 (E.1.1.) |
| **USA** *(n=5)* |
| 1982: M13178 (Au) |
| 2006: AY504945 (NAN) |
| 2007: FW377254 |
| 2008: FJ591158 (KU1) |
| **Belgium** *(n=2)* |
| 2000: DQ293995 (C39) |
| 2002: FN598217 (BB19) |
| **England** *(n=3)* |
| 1973: M24682 (Wi or PVBPRO) |
| 1990: AF162273 (HV) |
| 1990: NC_000883 (J35) or AY386330 |
| 1993: Z68146 (Stu) |
| **Vietnam** *(n=6)* |
| 2001: DQ225148-51 (KyMa, SN807, OsFr, AnTo) |
| 2001: DQ357064-5 (Vn147, Vn115) |
| **Netherlands** *(n=65)* |
| 2003: 14 isolates |
| 2004: 14 isolates |
| 2006: 14 isolates |
| 2008: 8 isolates |
| 2009: 15 isolates |
| JN211121 to JN211185 |
| **France** *(n=5)* |
| 2009: FN669502-4, FN669506-7 |
| **Brazil** *(n=15)* |
| 2007: KC013340, KC013343 |
| 2008: KC013312, KC013332 |
| 2009: KC013308, KC013314, KC013321, KC013324, KC013329, KC013331, KC013344 |
| 2010: KC013305, KC013325, KC013327, KC013333 |
| **Serbia** *(n=5)* |
| 2009: KR005643 (B1 M1-406 RS1) |
| 2011: KR005641 (3341 RS4), KR005642 (5248 RS6), KR005640 (1577 RS7) |
| 2012: KR005644 (M21 RS8) |

1B. Isolates used for codon selection analyses

| Coding region  (n = total number of sequences) | GenBank accession number |
| --- | --- |
| NS1/7.5 kDa  (n = 146) | AY386330, Z68146, FN598218, FN598217, FJ591158, AB030693, Z70560, DQ408301, AB030673, AB126271, M13178, KC013325, DQ225151, KC013343, AB126269, AF162273, AB126264, AB126263, AB126262, AY028232, AY028229, AF113323, KC013345, KC013308, AY028255, AY028238, AY028237, AY028234, KC013329, Z70599, Z70528, M24682, KC013316, KC013346, KC013324, KC013321, KC013312, KC013340, AY504945, AJ781038, AB030694, KC013327, DQ225150, KC013305, KC013344, KC013331, DQ293995, AB126266, KC013333, AB126268, KC013338, DQ225149, AB126267, AB126265, KC013314, JX027214, AB126270, DQ225148, JQ302818, JN638547, AJ781034, AJ781032, AJ781031, AJ781035, AJ781033, AF161226, AF161223, AF161224, AJ781036, AF161225, AJ781037, FN669502 - FN669504, FN669506, FN669507, JN211121 - JN211185, KR005640 - KR005644. |
| VP1/uVP1/VP2/9 kDa  (n = 214) | Z68146, AF161226, AF113323, FN598218, FN598217, AB030673, AB030693, FJ591158, AB126271, AY386330, AY028241, DQ408301, Z70560, M13178, KC013305, AB126264, Z70528, KC013324, AB126263, AB126262, KC013325, AB126269, AY028237, KC013329, KC013343, FJ429098, M24682, KC013321, Z70599, AB030694, AF162273, KC013340, DQ293995, KC013308, AY504945, KC013303, AJ781037, AB126267, KC013327, KC013313, AJ781036, KC013331, KC013312, HQ664953, DQ225151, EU478536, DQ225150, AB126268, AJ781032, AJ781031, AY661661, AY661660, KC013344, AJ781034, AY768535, AJ781038, AJ781035, DQ225149, KC013333, AB126265, HQ130278, DQ225148, AB126270, U38546, U53593, U53594, U31358, U38506 - U38518, EU478527 - EU478535, EU478537 - EU478555, EU478557 - EU478564, EU478566 - EU478589, FN669502 - FN669507, JN211121 - JN211185, KR005640 - KR005642, KR005644. |
| 11kDa  (n = 27) | KR005640 - KR005644, KR005639, Z70560, DQ408301, M13178, NC_000883, FW377254, FJ591158, DQ293995, FN598217, M24682, AF162273, Z68146, AY386330, KM393168, KM393167, KM393166, AY504945, KM393169, KM393165, KM393164, KM393163 |

**Supplementary Table S2 Codon selection analyses on 6 B19 genes**

Legend for Supplementary Table S2: ORF1 and ORF2 dataset consists of 146 and 214 sequences, respectively; ^a^GenBank accession numbers of sequences used for selection analyses are listed in Supplementary Table S2B; ^b^ Substitution model as determined by Datamonkey webserver for every alignment; ^c^ω values by different codon based methods, shown as normalized dN-dS with P values shown in brackets; ^d^codon position; codons in bold - positive selection detected by more than two methods

| **Data set^a^ (nt), model^b^** | **SLAC** ω (p) ^c^ | **FEL ω** (p) | **IFEL ω** (p) | **REL ω** (Log BF) | **MEME** β^+^ (p) |
| --- | --- | --- | --- | --- | --- |
| **NS1 (667 -2628), TrN93** |  |  |  |  |  |
| positively selected codons p<0.1 | 0 | 2 | 6 |  |  |
| 47^d^ (Y→L) | - | - | - |  | 8826.7 (1.4 x10^-4^) |
| 164 (C→L) | - | - | - |  | 10000 (3.24 x 10^-6^) |
| 183 (T→P, A) | - | - | 12.18 (0.083) |  | - |
| 195 (S→A, P) | - | - | 12.56 (0.052) |  | - |
| 279 (T→A) | - | - | 11.91 (0.085) |  | - |
| 509 (I→V) | - | - | 13.12 (0.025) |  | - |
| 545 (I→V) | - | - | 13.11 (0.025) |  | - |
| **554 (F →S, L)** | - | 11.03 (0.048) | 20.09 (0.023) |  | 3.78 (0.074) |
| 563 (V→A) | - | 7.48 (0.08) | - |  | - |
| negatively selected codons p<0.1 | 56 | 119 | 39 |  |  |
| mean dN/dS [Log (L)] | 0.150 [-7274.55] |  |  |  |  |
| Confidence interval CI, 95% | [0.128-0.174] |  |  |  |  |
| substitutions/site | 0.012±0.004 |  |  |  |  |
| **7.5 kDa, (2084 -2308), HKY85** |  |  |  |  |  |
| positively selected codons p<0.1 | 0 | 0 | 1 | 0 | 0 |
| 13 (Q→R) | - | - | 113.95 (0.06) | - | - |
| negatively selected codons p<0.1 | 1 | 5 | 0 | 2 |  |
| mean dN/dS [Log (L)] | 0.297 [-507.36] |  |  | 0.92 |  |
| Confidence interval CI, 95% | [0.154-0.509] |  |  |  |  |
| substitutions/site | 0.006±0.004 |  |  |  |  |
| **VP1 (2624 -4851), TrN93** |  |  |  |  |  |
| positively selected codons p<0.1 | 1 | 5 | 2 |  | 7 |
| **4 (E→N, K)** | - | 4.88 (0.08) | - |  | 16.20 (0.023) |
| **12 (D→N)** | 0.57 (0.08) | 4.66 (0.03) | 4.00 (0.07) |  | 5.54881 (0.071) |
| 30 (V→L) |  |  |  |  | 38.68 (0.081) |
| 61 (D→N) | - | 1.72 (0.07) | - |  | - |
| **107 (D→N)** | - | 2.87 (0.08) | 5.97 (0.03) |  | - |
| 129 (N→A) | - | - | - |  | 237.64 (6.70 x10^-4^) |
| 532 (S→F) | - | - | - |  | 461.85 (3.39 x10^-3^) |
| 533 (N→S) | - | 2.65 (0.09) | - |  | - |
| 548 (TCT→AGT) | - | - | - |  | 67.93 (0.048) |
| 604 (E→N) | - | - | - |  | 112.21 (0.003) |
| negatively selected codons p<0.1 | 170 | 287 | 143 |  |  |
| mean dN/dS [Log (L)] | 0.087 [-11694.8] |  |  |  |  |
| Confidence interval CI, 95% | [0.076-0.099] |  |  |  |  |
| substitutions/site | 0.014±0.007 |  |  |  |  |
| **uVP1 (2624 -3305), HKY85** |  |  |  |  |  |
| positively selected codons p<0.1 | 1 | 2 | 2 |  | 3 |
| **4 (E→N, K)** | - | - | 35.01 (0.07) |  | 54.09 (0.033) |
| **12 (D→N)** | 6.69 (0.10) | 23.89 (0.06) | - |  | 14.38 (0.078) |
| 21 (Q→E) | - | - | 16.68 (0.09) |  | - |
| 61 (D→N) | - | 8.72 (0.09) | - |  | - |
| 129 (N→A) | - | - | - |  | 94.86 (6.55 x10^-3^) |
| negatively selected codons p<0.1 | 14 | 32 | 15 |  |  |
| mean dN/dS [Log (L)] | 0.325 [-2345.55] |  |  |  |  |
| Confidence interval CI, 95% | [0.263-0.397] |  |  |  |  |
| substitutions/site | 0.007±0.004 |  |  |  |  |
| **VP2 (3305-4851), TrN93** |  |  |  |  |  |
| positive selected codon p<0.1 | 0 | 0 | 0 |  | 3 |
| 305 (S→F) | - | - | - |  | 271.60 (4.78 x10^-3^) |
| 321 (TCT→AGT) | - | - | - |  | 83.62 (0.03) |
| 377 (E→N) | - | - | - |  | 65.65 (4.11 x10^-3^) |
| negatively selected codons p<0.1 | 153 | 251 | 132 |  |  |
| mean dN/dS [Log (L)] | 0.055 [-8864.52] |  |  |  |  |
| Confidence interval CI, 95% | [0.046-0.065] |  |  |  |  |
| substitutions/site | 0.017±0.008 |  |  |  |  |
| **9kDa (2874 -3119), HKY85** |  |  |  |  |  |
| positive selected codon p<0.1 | 0 | 2 | 1 | 2 | 2 |
| **15 (A→T)** | - | 68.60 (0.04) | 85.84 (0.09) | 8.34 (3.23) | 12.61 (0.05) |
| **63 (L→W)** | - | 86.57 (0.03) | - | 8.03 (1.74) | 13.48 (0.05) |
| negatively selected codons p<0.1 | 1 | 5 | 0 | 1 | - |
| mean dN/dS [Log (L)] | 0.76 [-588.20] |  |  | 1.75 |  |
| Confidence interval CI, 95% | [0.494-1.10] |  |  |  |  |
| substitutions/site | 0.005±0.004 |  |  |  |  |
| **11kDa (4890 -5174), HKY85** |  |  |  |  |  |
| positively selected codons p<0.1 | 0 | 0 | 0 | 0 | 0 |
| negatively selected codons p<0.1 | 0 | 1 | 0 | 0 | 0 |
| mean dN/dS [Log (L)] | 0.464 [-471.42] |  |  |  |  |
| Confidence interval CI, 95% | [0.212-0.864] |  |  |  |  |
| substitutions/site | 0.007±0.004 |  |  |  |  |

Supplementary Table S3 RSCU values for two ORFs of B19 genome

|  | **ORF1** | | **ORF2** | |  | **ORF1** | | **ORF2** | |
| --- | --- | --- | --- | --- | --- | --- | --- | --- | --- |
| **Codon** | **Count** | **RSCU** | **Count** | **RSCU** | **Codon** | **Count** | **RSCU** | **Count** | **RSCU** |
| UUU(F) | 29.4 | 1.86 | 29.2 | 1.95 | UAU(Y) | 11.9 | 1.26 | 23.4 | 1.34 |
| UUC(F) | 2.2 | 0.14 | 0.8 | 0.05 | UAC(Y) | 7.1 | 0.74 | 11.6 | 0.66 |
| UUA(L) | 17.8 | 1.9 | 37.1 | 3.69 | UAA(*) | - | - | - | - |
| UUG(L) | 12.2 | 1.31 | 5.8 | 0.58 | UAG(*) | - | - | - | - |
| CUU(L) | 6.2 | 0.66 | 7.2 | 0.72 | CAU(H) | 10 | 1.43 | 15.3 | 1.13 |
| CUC(L) | 4.6 | 0.49 | 0 | 0 | CAC(H) | 4 | 0.57 | 11.7 | 0.87 |
| CUA(L) | 11.2 | 1.2 | 7.1 | 0.71 | CAA(Q) | 11.4 | 1.27 | 23.9 | 1.14 |
| CUG(L) | 4.1 | 0.44 | 3 | 0.3 | CAG(Q) | 6.6 | 0.73 | 18.1 | 0.86 |
| AUU(I) | 19.8 | 1.93 | 17.8 | 1.91 | AAU(N) | 20.3 | 0.99 | 21.4 | 1.23 |
| AUC(I) | 3 | 0.29 | 0.2 | 0.03 | AAC(N) | 20.7 | 1.01 | 13.3 | 0.77 |
| AUA(I) | 8 | 0.78 | 10 | 1.07 | AAA(K) | 26.2 | 1.42 | 29.1 | 1.49 |
| AUG(M) | 10.3 | 1 | 13 | 1 | AAG(K) | 10.8 | 0.58 | 9.9 | 0.51 |
| GUU(V) | 14.9 | 1.05 | 14.9 | 1.39 | GAU(D) | 18 | 1.13 | 13.6 | 0.82 |
| GUC(V) | 3.1 | 0.22 | 1.8 | 0.17 | GAC(D) | 14 | 0.87 | 19.6 | 1.18 |
| GUA(V) | 21.8 | 1.53 | 14.7 | 1.37 | GAA(E) | 21.5 | 1.4 | 26.8 | 1.41 |
| GUG(V) | 17.2 | 1.21 | 11.4 | 1.06 | GAG(E) | 9.1 | 0.6 | 11.1 | 0.59 |
| UCU(S) | 10.9 | 1.28 | 17.9 | 1.58 | UGU(C) | 14.1 | 1.34 | 1.1 | 0.43 |
| UCC(S) | 2.3 | 0.27 | 5 | 0.44 | UGC(C) | 6.9 | 0.66 | 3.9 | 1.57 |
| UCA(S) | 2.2 | 0.25 | 9.1 | 0.8 | UGA(*) | - | - | - | - |
| UCG(S) | 1 | 0.11 | 0 | 0 | UGG(W) | 19 | 1 | 11 | 1 |
| CCU(P) | 8.3 | 1.07 | 16 | 1.16 | CGU(R) | 2 | 0.74 | 1.9 | 0.69 |
| CCC(P) | 7.7 | 0.99 | 15.4 | 1.12 | CGC(R) | 2.9 | 1.09 | 4 | 1.42 |
| CCA(P) | 13 | 1.67 | 20.3 | 1.48 | CGA(R) | 1.5 | 0.56 | 0 | 0.01 |
| CCG(P) | 2.1 | 0.27 | 3.3 | 0.24 | CGG(R) | 0.5 | 0.2 | 1 | 0.36 |
| ACU(T) | 18.8 | 1.51 | 21.6 | 1.76 | AGU(S) | 16.9 | 1.98 | 24.2 | 2.14 |
| ACC(T) | 13.2 | 1.06 | 8.4 | 0.69 | AGC(S) | 18.1 | 2.11 | 11.7 | 1.04 |
| ACA(T) | 16.8 | 1.35 | 18.1 | 1.47 | AGA(R) | 6 | 2.24 | 7.9 | 2.8 |
| ACG(T) | 1 | 0.08 | 1 | 0.08 | AGG(R) | 3.1 | 1.16 | 2.1 | 0.73 |
| GCU(A) | 14.9 | 1.61 | 17.4 | 1.42 | GGU(G) | 6.2 | 0.48 | 15.4 | 0.95 |
| GCC(A) | 8 | 0.87 | 13.6 | 1.11 | GGC(G) | 8 | 0.62 | 5.1 | 0.31 |
| GCA(A) | 14.1 | 1.52 | 17.9 | 1.46 | GGA(G) | 17.9 | 1.39 | 29.4 | 1.81 |
| GCG(A) | 0.1 | 0.01 | 0.2 | 0.01 | GGG(G) | 19.3 | 1.5 | 15.1 | 0.93 |

**Supplementary Table S4 List of sequencing primers**

**Legend for Supplementary Table S4: ^a^nucleotides numbered according to the B19 reference sequence NC_000883.2.**

| name | sequence | location^a)^ | reference |
| --- | --- | --- | --- |
| NSNI^G1^: 1-F | 5’-GACGTAATTGTCCGCCATCT-3’ | 297-316 | Schneider et al., 2008^36^ |
| 1N-F | 5’-TACCGGAAGTCCCGCCTAC-3’ | 319-337 | Schneider et al., 2008^36^ |
| NV1-F | 5’- AACTAACAGGTATTTATACT -3’ | 578-597 | designed for this study |
| G1-F | 5’-TAGATACCTGTATTTCTGCTA-3’ | 1097–1117 | designed for this study |
| 1N-R | 5’-ATGGCAAGCTCCCCTTCTAA-3’ | 1121-1140 | Schneider et al., 2008^36^ |
| NSNI^G1^: 1-R | 5’-GCTAGACTCCCCAGCATCAC-3’ | 1184-1203 | Schneider et al., 2008^36^ |
| G1-R | 5’-ATAGTTTGAAACTTTATGCTA-3’ | 1251–1271 | designed for this study |
| P3-F | 5’-TGGATTGATAAAAAATGTGG -3’ | 1552-1571 | Koppelman et al., 2007^11^ |
| NV2-R | 5’-GCCATTGCCAAGTTTGTTTT-3’ | 1615-1634 | designed for this study |
| NS-F | 5’-TGCAGATGCCCTCCACCCA-3’ | 2085-2103 | Servant et al, 2002^13^ |
| NSn-R | 5’-TGCTTTCACTGAGTTCTTC-3’ | 2167-2185 | designed for this study |
| PV-2-(f) | 5’-GCTTGGTATAATGGATGGAA-3’ | 2482-2501 | Koppelman et al., 2007^11^ |
| PV-3(r) | 5’-CCAGACAGGTAAGCACATTT-3’ | 2584-2603 | Koppelman et al., 2007^11^ |
| VP1-R | 5’-TTGGCTATACCTAAAGTCAT-3’ | 3081-3100 | Servant et al, 2002^13^ |
| NV3-R | 5’-TTATAATGGTGCTCTGGGTCA-3’ | 3448-3468 | designed for this study |
| NV4-F | 5’-CCCAAGCATGACTTCAGTTA-3’ | 3298-3317 | designed for this study |
| NV5-F | 5’-TCAGCTTTTAGGTACAGGAGG-3’ | 3961-3981 | designed for this study |
| NV4-R | 5’-AAGTGTTGACTGCAGCCCTC-3’ | 4031-4050 | designed for this study |
| VPCII^G1^: 2-F | 5’-GCAAGGAGTGGGTAGATTTCC-3’ | 4457-4477 | Schneider et al., 2008^36^ |
| VPCII^G1^ : 2-R | 5’-ACGGTGGGGAGTGTTTACAA-3’ | 4919-4939 | Schneider et al., 2008^36^ |
| 2N-F | 5’-CAGGGTTTAAACATGCACACC-3’ | 4457-4477 | Schneider et al., 2008^36^ |
| 2N-R | 5’-CCACAATTCTTCAGGCTTTTC-3’ | 4919-4939 | Schneider et al., 2008^36^ |
| VPintIV^G1^: 3-F | 5’-CCAGCTGTGGAGTAAAATTCCAAAT-3’ | 4576-4600 | Schneider et al., 2008^36^ |
| NV5-R | 5’-CCCAATTTAAATGTCATAGT-3’ | 4769-4788 | designed for this study |
| 3N-F | F 5’-AGTTTGCAGCCTTAGGAGGA-3’ | 4623-4642 | Schneider et al., 2008^36^ |
| 3N-R | 5’-CGGCATCTGATTTGGTGTC-3’ | 5228-5246 | Schneider et al., 2008^36^ |
| VPintIV^G1^: 3-R | 5’-ACCGGAAGTCCCGCCTAC-3’ | 5260-5277 | Schneider et al., 2008^36^ |
